# Supramolecular Gel Formation Based on Glycolipids Derived from Renewable Resources

**DOI:** 10.3390/gels4010001

**Published:** 2017-12-24

**Authors:** Krishnamoorthy Lalitha, Kandasamy Gayathri, Yadavali Siva Prasad, Rajendhiran Saritha, A. Thamizhanban, C. Uma Maheswari, Vellaisamy Sridharan, Subbiah Nagarajan

**Affiliations:** 1 Organic Synthesis Group, Department of Chemistry and CeNTAB, School of Chemical and Biotechnology, SASTRA Deemed University, Thanjavur-613401, Tamil Nadu, India; lalitha@scbt.sastra.edu (K.L.); gayathri.ks86@gmail.com (K.G.); siva.sathyabama@gmail.com (Y.S.P.); rtmpsaritha@gmail.com (R.S.); tamizhan.1992@gmail.com (A.T.); umamaheswaric@scbt.sastra.edu (C.U.M.); vesridharan@gmail.com (V.S.); 2 Department of Chemistry and Chemical Sciences, Central University of Jammu, Rahya-Suchani (Bagla), District-Samba, Jammu-181143, Jammu and Kashmir, India

**Keywords:** glycolipid, cashew nut shell liquid, hydrogel, sophorolipid, molecular self-assembly, soft materials

## Abstract

The potential applications of self-assembled supramolecular gels based on natural molecules encouraged the researchers to develop a versatile synthetic method for their structural analogues. Herein, we report a facile synthesis of glycolipid from renewable resources, cashew nut shell liquid,d and d-glucose in good yield. Gelation behavior of these glycolipids were studied in a wide range of solvents and oils. To our delight, compound **5b** formed a hydrogel with Critical gelator concentration (CGC) of 0.29% *w*/*v*. Morphological analysis of the hydrogel depicts the formation of twisted fibers with an entangled network. Formation of a twisted fibrous structure was further identified by CD spectral studies with respect to temperature. The molecular self-assembly assisted by hydrogen bonding, hydrophobic, and π–π stacking interactions were identified by X-ray diffraction (XRD) and FTIR studies. Rheological analysis depicted the mechanical strength and stability of the hydrogel, which is crucial in predicting the practical applications of supramolecular soft materials.

## 1. Introduction

Nature provides plenty of prospects for constructing structural and functional materials from the various raw materials, such as carbohydrates, nucleotides, and proteins, which perform unique and complex functions [1]. Thus, design and development of self-assembled supramolecular soft materials from low molecular weight compounds have acquired remarkable research interest because of their tendency to build a variety of architectures via non-covalent interactions, like π–π stacking, hydrogen bonding, dipole-dipole interaction, and van der Waals forces [2,3,4,5]. Generally, these supramolecular soft materials display responsive behavior to various external stimuli, such as temperature, light, pH, ions, mechanical stress, ultrasound, and enzymes, which, when harnessed effectively, can produce functional materials for applications such as drug carriers, enzyme immobilization, sensors, soft optical devices, dye-sensitized solar cells, templating components for inorganic or organic nanostructures, cell scaffolds, and wound healing [6,7,8,9,10]. Recently, a wide variety of small molecules and polymers of natural and petrochemical origin were utilized for the generation of supramolecular soft-materials [11,12]. Among the reported biocompatible natural raw materials, carbohydrates, also called a chiral pool, have become an obvious choice to construct soft materials due to its eco-friendliness, cost effectiveness, biodegradability, and structural diversity. Furthermore, the construction of multifunctional architectures using sugar-derived low molecular weight gelators (LMWGs) has gained much interest because of their interaction with protein in various biological phenomena, such as blood coagulation, immune response, inflammation, and intra-cellular signal transfer [13,14,15,16,17,18]. In light of potential applications of carbohydrates, Shimizu and co-workers have reported the molecular self-assembly of various sugar-based lipids and their structural effects [19,20]. John and co-workers have enzymatically synthesized a bio-based amphiphile from a natural sugar-based molecule, amygdalin, and demonstrated the enzyme triggered the release of the natural drug curcumin [21]. Sophorolipids are microbial glycolipids known to self-assemble into various supramolecular architectures and are ubiquitous constituents of biological surfactants that perform functions based on the complex structure-dependent interactions [22,23]. A detailed comprehensive review on sophorolipid depicted the potential application in the food industry and biomedical field [24]. Even though sophorolipids show excellent biological properties, such as antiviral, antimicrobial, and anticancer properties, they displayed several limitations, such as solubility, stability of *O*-glycosidic bonds, and hydrolysis of ester bonds, which makes the molecule unfavorable for oral administration [25]. As the carbohydrates are amenable to prepare the broad classes of self-assembled soft materials with a wide range of applications, we desired to synthesize an analogue of sophorolipid directly from glucose and cashew nutshell liquid (CNSL). Among the wide range of renewable resources reported, CNSL is considered an important by-product of the cashew nut industry. More than 32% of the cashew shell is CNSL and the key constituent is cardanol, a bio-based non-isoprene lipid, consisting of phenolic lipids: 5% of 3-(*n*-pentadecyl)phenol (3-PDP), 50% of 3-(8*Z*-pentadecenyl)phenol, 16% of 3-(8*Z*,11*Z*-pentadecadienyl)phenol and 29% of 3-(8*Z*,11*Z*,14-pentadecatrienyl)phenol [26,27]. The presence of varying degree of *cis*- double bonds and an odd number of hydrocarbons in cardanol is considered as a unique feature, which can render a wide variety of functional materials [28,29,30,31,32,33,34]. In the present study, we report the synthesis and self-assembly of renewable resource-derived glycolipids, which is an analogue of sophorolipid. Our investigation furnishes an insight on the influence of molecular structure on supramolecular self-assembly.

## 2. Results and Discussion

**Figure 1:**
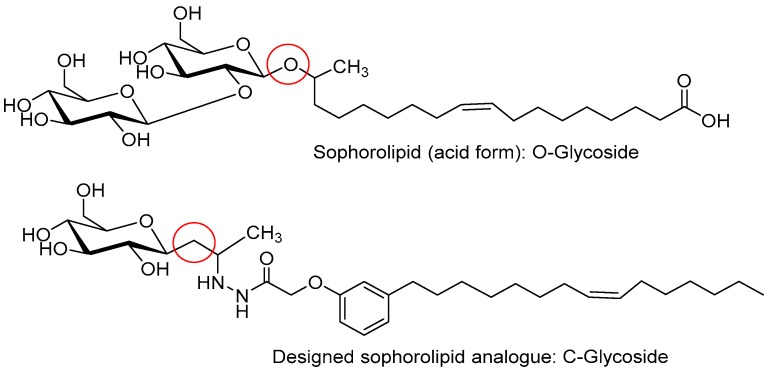
Molecular structure of sophorolipid and the designed saphorolipid analogue.

By considering the potential applications of sophorolipids, in this report we have synthesized an analogue of sophorolipid, C-glycolipid, from readily-available renewable resources, cardanol derivatives, and β-glycosidic ketone, by adopting simple synthetic protocols. The molecular structure of sophorolipid and the designed analogue is given in Figure 1.

**Scheme 1:**
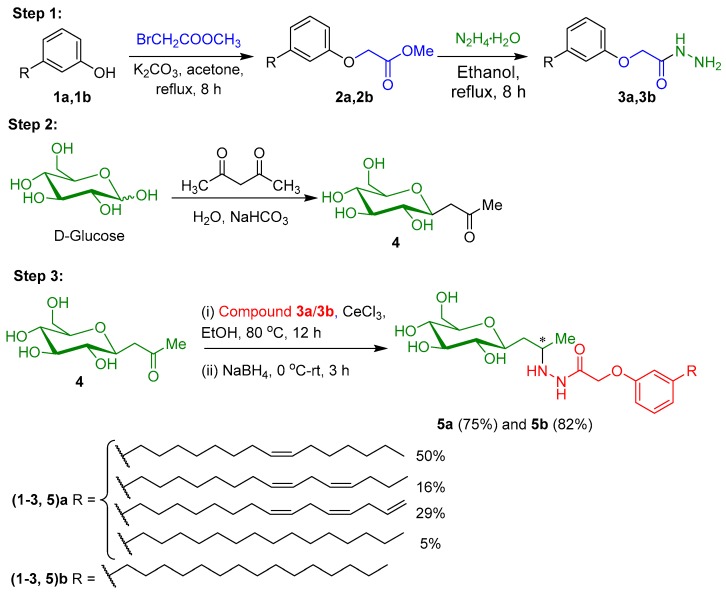
Synthesis of C-glycolipids **5a** and **5b**.

In the first step, synthesis of 2-(3-alkylphenoxy)acetohydrazides (**3a** and **3b**) involves the formation of the corresponding ester (**2a** and **2b**) from cardanol derivatives (**1a** and **1b**), followed by the reaction with hydrazine hydrate in ethanol under reflux condition (Scheme 1). The appearance of the proton signal at *δ* = 3.93, 4.57, 6.69–7.22, and 7.75 ppm correspond to –NH_2_, –O–CH_2_–, aromatic hydrogens, and –NH protons, as compound **3a** confirms the product formation. In ^13^C NMR spectra of compound **3a**, the carbonyl group appears at *δ* = 168.8 ppm and –O–CH_2_– at *δ* = 66.9 ppm, further confirming the product formation [35]. The second step involves the synthesis of β-C-glycosidic ketone **4** by Knoevenagel condensation of 2,4-pentadienone with d-glucopyranose using the reported procedure [36] (Scheme 1). The introduction of the ketone functionality at the saccharide moiety enables the possibility of further derivatization with amines, such as hydroxylamines, aromatic amines, aliphatic amines, and hydrazines. In the final step, 2-(3-alkylphenoxy)acetohydrazides (**3a** and **3b**) reacts with β-C-glycosidic ketone **4** in the presence of cerium chloride as a catalyst and ethanol under reflux conditions for about 12 h, resulting in the formation of the hydrazone intermediate, which, on subsequent reduction by sodium borohydride, furnished the corresponding C-glycolipids (**5a** and **5b**) in 75% and 82%, respectively (Scheme 1).

Newly-synthesized glycolipids, **5a** and **5b**, were characterized using NMR spectral techniques. Though the precursor saccharide, d-glucose, can exist as a mixture of both α and β anomers, the corresponding C-glycoside, **4**, exists only in the β-anomeric form [37], as confirmed by NMR spectral analysis (Figure S5). Further chemical modification of compound **4** retained the conformation of the sugar moiety. The ^1^H NMR spectra of compound **5b** displayed peaks at *δ* 8.4 ppm, *δ* 4.5 ppm, and *δ* 4.4 ppm corresponding to −NH and −OH protons. Moreover, a methyl peak appeared at *δ* 2.1 ppm for the hydrazone intermediate shifted to *δ* 1.03 ppm when reduced with NaBH_4_, which is directly indicative of product formation. In the ^13^C NMR spectrum, the aromatic skeletal carbons appear between 110–158 ppm, carbonyl carbon appears at 162 ppm, saccharide skeletal carbons are observed in the region of 60–79 ppm, and the methyl group (−CH−CH_3_) appears at 11 ppm. Since these glycolipids were amphiphilic in nature, we could not observe their well-resolved spectra; instead, a broadening in signals occurred because of the instant self-assembly of these molecules in solvent.

### 2.1. Gelation Studies

Recently, Xu and co-workers have presented a comprehensive summary of supramolecular hydrogelators as smart biomaterials and emphasized their potential applications in cell cultures, tissue engineering, cell behavior, and imaging [38]. Generally, these self-assembled supramolecular gel materials were formed through non-covalent interactions, such as π–π stacking, hydrogen bonding, and van der Waals interactions. The broad scope of low molecular weight gels has created interest in us to design and develop the glycolipid-based gels. The gelation abilities of the glycolipids **5a** and **5b** were examined in various solvents by the “stable to inversion in a test tube” method (Table 1) [36,37]. Compound **5b**, having a saturated alkyl tail, showed excellent gelation ability in ethanol-water (0.2:1) with a CGC of 0.29% (*w*/*v*), whereas partial gelation occurred in the case of **5a**, which is attributed to the existence of a kink in the hydrophobic part of **5a**. Compounds **5a** and **5b** did not show gel formation in organic solvents and water, whereas partial gelation was observed in the case of paraffin oil. However, the use of a mixture of solvents, such as Dimethyl sulfoxide-H_2_O (DMSO-H_2_O), *N,N*-Dimethylformamide-H_2_O (DMF-H_2_O), MeOH-H_2_O, and EtOH–H_2_O in a ratio of 1:5 resulted in gel formation. Thus, the use of hydrophilic organic solvent as a co-solvent facilitated the molecular self-assembly. The gel formed by **5b** in the ethanol-water mixture (0.29% *wt*/*v*) was found to be thermoreversible in nature and displayed a gel-to-sol transition at 48 °C. This result clearly indicates that a small change in molecular structure, such as the existence of a kink in the hydrophobic part, suppresses the molecular self-assembly phenomenon, which resulted in the formation of a partial gel or remains as a solution.

### 2.2. Morphological Analysis of the Gel

**Figure 2:**
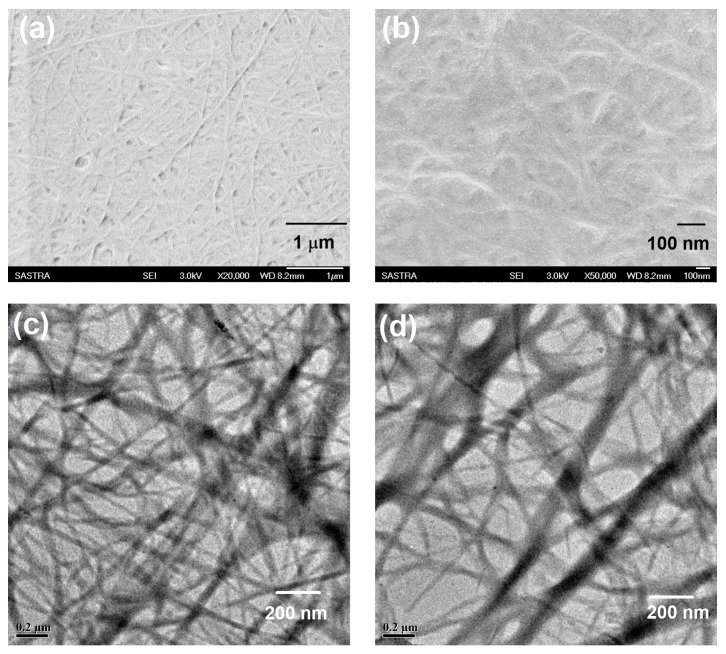
(**a**,**b**) Field Emission Scanning Electron Microscope (FESEM) image (**c**,**d**) and Field Emission Transmission Electron Microscope (FETEM) image of gel prepared from compound **5b** in water-ethanol (1:5).

In order to obtain further insight into the aggregation mode at the nanoscale, the morphology of the gel prepared from compound **5b** in water-ethanol was examined using Field Emission Scanning Electron Microscope (FESEM) and Field Emission Transmission Electron Microscope (FETEM) (Figure 2). When a slice of the gel was carefully taken and subjected for morphological analysis using FESEM, a well-developed network structure composed of fibrous aggregates with lengths of 100–200 nm was observed (Figure 2). FETEM analysis clearly revealed the formation of a highly-entangled twisted fibrous network with a larger void volume. The existence of nanofibers with greater void volumes can trap more solvent in it, as documented by the FESEM and FETEM analysis.

### 2.3. Absorption and CD Spectral Studies

**Figure 3:**
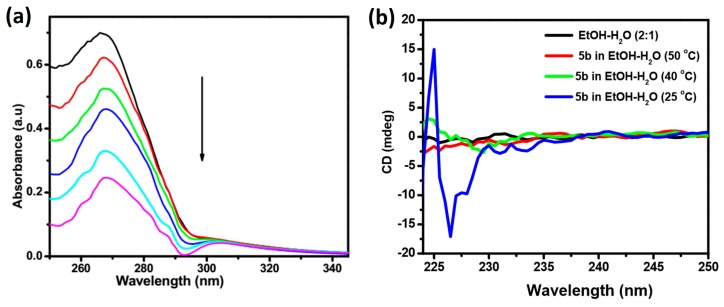
(**a**) UV-VIS titration of gelator **5b** dissolved in ethanol (1 × 10^−5^ M) with water. In the titration experiment, the direction of the arrow shows the response of absorbance/wavelength with the piecemeal addition of 100 μL of water. Two milliliters of the initial volume of solution (1 × 10^−5^ M) was taken for titration experiments; and (**b**) CD spectra of gelator **5b** at variable temperature (1 × 10^−5^ M).

The process involved in molecular self-assembly of **5b** was further confirmed by the UV-VIS absorption spectral technique. In the UV-VIS titration of **5b** in ethanol vs. water, the intensity of the absorbance band at 266 nm gradually decreases with the sequential addition of 100 μL of water. The addition of water effected the molecular self-assembly process via the weak forces, which substantially reduces the absorbance of compound **5b** (Figure 3a). Circular dichroism (CD) is a valuable technique to probe the chirality of supramolecular assembly. Induction of the Cotton effect on molecular self-assembly of our system can be probed by performing the experiment at variable temperature. As shown in Figure 3b, ethanol-water (1:5) mixture and compound **5b** in ethanol-water (1:5) at 50 °C displayed CD silent, which attributes the absence of molecular aggregation. The decrease in temperature to 40 °C resulted in the initiation of molecular aggregation, resulting in the appearance of the CD spectra. However, at 25 °C, supramolecular aggregation occurred resulting in the formation of twisted fibers showing a positive Cotton effect at 225 nm and a negative effect at 226 nm. The presence of intermolecular hydrogen bonding, π–π stacking, and the hydrophobic interaction of alkyl chains effected left-landed twist molecular chirality [39].

### 2.4. FTIR Studies

**Figure 4:**
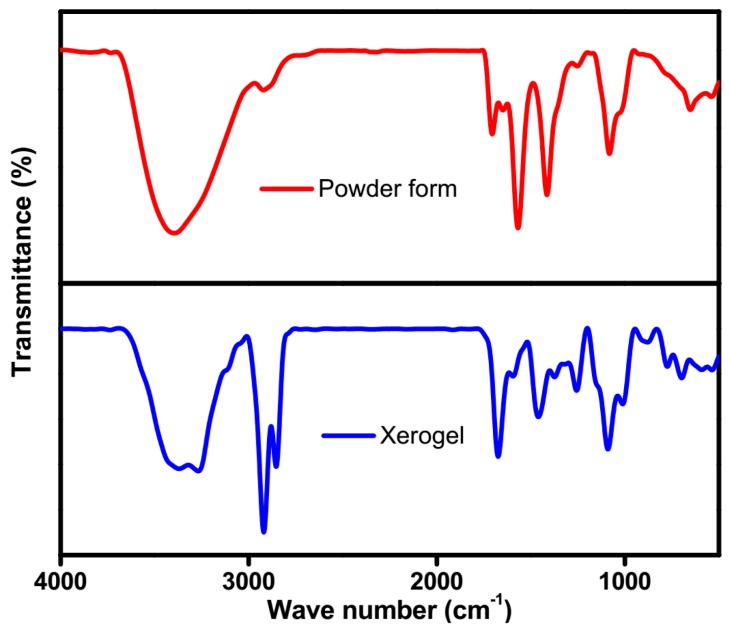
FTIR spectra of compound **5b** in powder form (**red**) and xerogel (**blue**).

FTIR spectroscopy is a potential tool to identify the existence of non-covalent interactions in the self-assembled state. IR spectra of compound **5b** in powder form and xerogel were recorded to determine the non-covalent interactions (Figure 4). The spectrum of the gelator in powder form exhibits peaks at 3396 cm^−1^ (NH stretching frequency), 1710 and 1650 cm^−1^ (symmetric and asymmetric stretching frequency carbonyl group), and 1568 cm^−1^ (–NH, amide) ascribed to non-hydrogen bonded NH and carbonyl stretching frequencies. In xerogel, the NH band shifted to lower wave numbers (3367 and 3274 cm^−1^) and the carbonyl and CONH shifted to 1673 and 1595 cm^−1^ (symmetric and asymmetric stretching frequency carbonyl group), and 1459 and 1365 cm^−1^ (–NH, amide), respectively, confirming the involvement of hydrogen bonding between carbonyl and amide –NH groups [40]. The peak at 2922 cm^−1^ (–CH_2_ stretching frequency) of the gelator in powder form was shifted to 2919 and 2854 cm^−1^ (symmetric and asymmetric stretching vibrations of –CH_2_), which suggested that the alkyl groups of **5b** were organized in the self-assembled nanostructure [41].

### 2.5. XRD Studies

**Figure 5:**
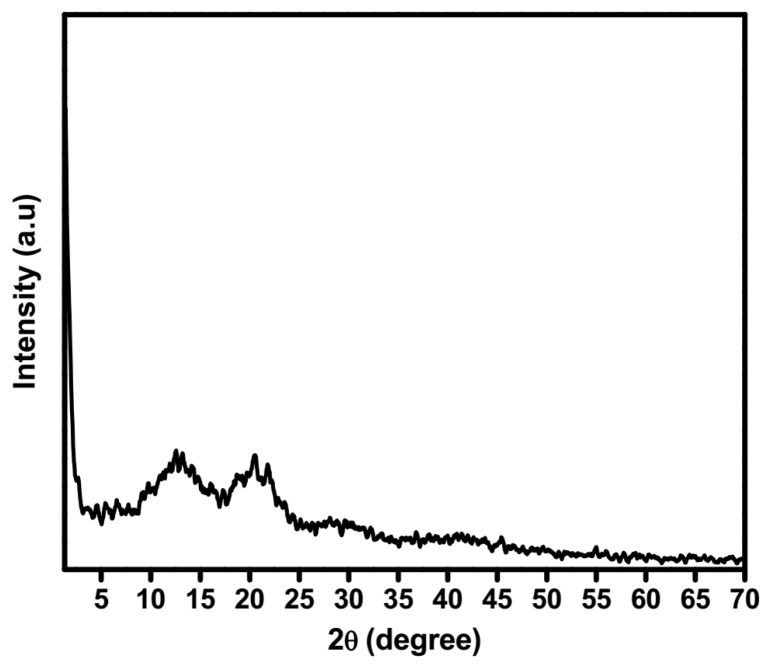
X-ray diffraction (XRD) pattern of the gel formed from compound **5b**.

X-ray diffraction (XRD) is used for identifying the molecular packing of gelators in the gel and the gelation mechanism of low molecular-weight gelators. XRD spectra of xerogel obtained from compound **5b** showed the reflection peak at 2θ = 2.8° (3.13 nm), 4.6° (1.93 nm), 5.3° (1.68 nm), 6.4° (1.4 nm), 12.4° (0.71 nm), 13.3° (0.67 nm), 20.7° (0.43 nm), and 21.9° (0.41 nm), which are almost exactly 1:1/2:1/5:1/3 (Figure 5). The appearance of a series of sharp peaks suggest that long alky chain groups form highly-ordered layer packing by the interdigitated hydrophobic interaction [14].

### 2.6. Rheological Studies

**Figure 6:**
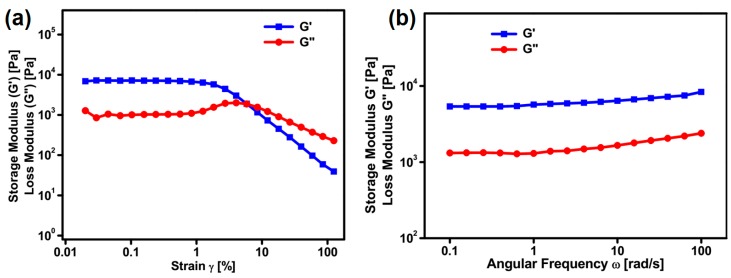
(**a**) Strain amplitude and (**b**) angular frequency dependence of G’ and G” of the hydrogel prepared from compound **5b**.

Rheological studies furnish the elastic behavior and flow characteristics of the structured material, which could be inferred from the storage modulus (G’) and loss modulus (G”) [42]. Generally, a detailed rheological investigation of self-assembled materials allow researchers to classify the direct practical application. The variation of storage modulus (G’) and loss modulus (G”) of the hydrogel formed from compound **5b** was analyzed by a frequency sweep experiment at a constant strain of 1% at room temperature (Figure 6). Across the entire range of the frequency sweep, the G’ value is greater than G”, confirming the mechanical strength of the hydrogel towards external forces (Figure 6). The viscoelastic nature of the self-assembled soft material with respect to strain could be identified by a strain amplitude experiment. Generally, a soft material retains its structural integrity until it reaches a critical strain level (γ_c_), and beyond γ_c_ it loses its structural feature and the material behaves in a non-linear fashion, which could be identified by the declining value of G’. In the strain amplitude experiment, G’ and G” remains constant up to a certain point. After that, a gradual drop of G’ and G” was observed and crossover occurs between them. The crossover point is conceived as the critical strain (γ_c_) of a hydrogel, which was found to be 6.1 (G’ = G” = 1951 Pa) (Figure 6). Rheological studies clearly depict the strength of the structured material, which is convenient to use for practical applications.

## 3. Conclusions

In summary, we have designed and synthesized sophorolipid analogues from renewable resources, cashew nut shell liquid, and d-glucose in good yield using a simple protocol. The gelation ability of glycolipid depends on the molecular structure; especially, a small tuning of the molecular structure drastically alters the self-assembly behavior. The morphological investigation implies the formation of a twisted fibrous network via the molecular self-assembly assisted by the co-operative effect of hydrogen bonding, hydrophobic, and π–π stacking interactions. The formation of twisted fibers was further confirmed by CD spectral studies at different temperatures. Rheological investigation furnishes the mechanical strength and stability of the hydrogel. The reported thermo-reversible glycolipid-based gel can be potentially used for practical applications. Further detailed investigations on drug encapsulation and release studies are in progress.

## 4. Materials and Methods

### 4.1. General Materials and Methods

All reagents and solvents required for the synthesis of glycolipids were purchased from Sigma Aldrich (St. Louise, MO, USA), Merck (Kenilworth, NJ, USA), Alfa aesar (Karlsruhe, Germany) and Avra chemicals (Hyderabad, India) and were used without further purification. All solvents were dried and freshly distilled before use. Solvents used for gelation studies are of AR grade. Pre-coated silica gel plates used in monitoring the progress of the reaction by thin-layer chromatography were purchased from Merck and visualized by UV detection or using sulfuric acid spray or molecular iodine. Column chromatography was performed on Silica Gel (100–200 mesh) purchased from Avra synthesis, India.

### 4.2. Characterization Methods

^1^H NMR and ^13^C NMR spectra were recorded on Bruker Avance 300 MHz NMR Spectrometer in either CDCl_3_ or DMSO-*d*_6_ or CDCl_3_ with few drops of DMSO-*d*_6_ at 298 K. Chemical shifts (*δ*) were reported in parts per million (ppm) with respect to internal standard TMS and coupling constants (*J*) are denoted in Hz. Proton multiplicity is assigned using the following abbreviations: singlet (s), doublet (d), triplet (t), quartet (q), multiplet (m). FT-IR spectra of the compound **5b** in powder and xerogel form were recorded using Perkin Elmer 100 FTIR Spectrometer in the spectral range of 4000 to 500 cm^−1^.

### 4.3. Synthesis

General procedure for the synthesis of 2-(3-alkylphenoxy)acetohydrazides, **3a** and **3b** 2-(3-alkylphenoxy)acetohydrazides, **3a** and **3b** were synthesized by following the literature procedure [32].

#### 4.3.1. Synthesis of 2-(3-Alkylphenoxy)acetohydrazides, **3a** and **3b**

To the solution of methyl 2-(3-alkylphenoxy)acetates, **2a** and **2b** (1.0 mmol) in ethanol, hydrazine hydrate (2.0 mmol) was added and refluxed for 12 h. After the completion of the reaction, as identified by TLC, the reaction mixture was cooled and the precipitated product was filtered and dried under *vaccum*. The crude product was further purified by recrystallization in ethanol.

**Compound 3a** Isolated as white amorphous solid; mp: 64–66 °C; yield = 82%. ^1^H NMR (300 MHz, CDCl_3_) *δ* = 7.75 (s, 1H), 7.22 (t, *J* = 7.8 Hz, 1H), 6.86 (d, *J* = 7.8 Hz, 1H), 6.76–6.69 (m, 2H), 5.38–5.02 (m, 2H), 4.57 (s, 2H), 3.93 (s, 2H), 2.58 (t, *J* = 7.8 Hz, 2H), 1.67–1.57 (m, 4H), 1.31–1.25 (m, 18H), 0.88 (t, *J* = 6.9 Hz*,* 3H); ^13^C NMR (75 MHz, CDCl_3_) *δ* = 168.8, 157.2, 145.1, 130.0, 129.5, 122.4, 114.8, 111.5, 66.9, 36.0, 32.6, 31.9, 31.8, 31.3, 29.7, 29.5, 29.4, 29.3, 29.2, 29.0, 27.2, 22.7, 14.1.

**Compound 3b** Isolated as white amorphous solid; mp: 90–92 °C; yield = 93%. ^1^H NMR (300 MHz, CDCl_3_) *δ* = 7.75 (br, 1H), 7.26–7.21 (m, 1H), 6.86 (br, 1H), 6.75–6.68 (m, 2H), 4.57 (s, 2H), 3.93 (br, 2H), 2.61–2.56 (m, 2H), 1.69–1.55 (m, 2H), 1.44–1.26 (m, 24H), 0.88 (m, 3H); ^13^C NMR (75 MHz, CDCl_3_) *δ* = 168.8, 157.1, 145.2, 129.5, 122.4, 114.7, 111.4, 66.9, 35.9, 31.9, 31.4, 29.7, 29.3, 22.7, 14.1.

#### 4.3.2. Synthesis of 1-(α-d-Glucopyranosyl)-propan-2-one 4

To a solution of d-glucose (1.80 g, 10.0 mmol) in 8:2 water-THF (10.0 mL) were added NaHCO_3_ (3.36 g, 40.0 mmol) and 2,4-pentadienone (2.1 mL, 20.0 mmol). After stirring at 90 °C for about 24 h, followed by concentration to dryness under reduced pressure and fractionation by column chromatography with 4:6 (CHCl_3_:MeOH) resulted in product, **4** as colorless syrup. Yield = 85% [36].

#### 4.3.3. Synthesis of Glycolipids **5a** and **5b**

To a suspension of β-C-glycosidic ketone **4** (1.0 mmol) dissolved in ethanol, hydrazides (1.2 mmol) and cerium chloride (catalytic amount) was added and heated the entire content at 60 °C for about 12 h. After completion of reaction as identified using Thin-Layer Chromatography (TLC), the reaction mixture was cooled to rt, then sodium borohydride (3 equiv.) were added into the mixture and stirred at room temperature until the complete reduction occurs. The reaction mixture was cooled, added to water to dissolve solid residues and extracted with Dichloromethane (DCM). The organic layer was washed with water and concentrated by evaporation under reduced pressure. The product was purified using silica gel column (100–200 mesh) using 10% chloroform-methanol mixture.

**Compound 5a** ^1^H NMR (300 MHz, DMSO-*d*_6,_ Figure S1) *δ* = 8.01 (s, 1H, –NH), 7.19–7.13 (m, 1H, Ar–H), 6.79–6.75 (m, 3H, Ar–H), 5.36-5.33 (m, 2H, Sac–H, Alk–H), 4.98 (m, 1H, Sac–H), 4.54 (s, 2H), 3.75 (d, *J* = 11.7 Hz, 2H, Sac–H), 3.65–3.63 (m, 2H, Sac–H), 3.51 (s, 2H, Sac–H), 3.38–3.29 (m, 2H, Sac–H), 2.68 (s, 2H), 1.99–1.95 (m, 5H), 1.86 (s, 2H), 1.59 (br s, 3H), 1.30–1.26 (m, 16H), 0.89 (d, *J* = 6.0 Hz, 3H). ^13^C NMR (75 MHz, DMSO-*d*_6,_ Figure S2) *δ* = 168.6, 156.2, 143.8, 138.2, 128.9, 128.8, 120.9, 113.1, 110.6, 76.3, 70.9, 69.1, 69.0, 65.7, 64.3, 51.2, 36.4, 34.9, 32.8, 30.9, 30.9, 30.8, 30.6, 30.4, 30.3, 28.8, 28.7, 28.6, 28.5, 28.4, 28.3, 28.2, 27.9, 26.2, 24.6, 21.7, 13.1.

**Compound 5b** ^1^H NMR (300 MHz, CDCl_3_, Figure S3) *δ* = 8.4 (s, 1H, –NH), 7.14–7.06 (m, 1H, Ar–H), 6.80–6.61 (m, 3H, Ar–H), 4.59 (s, 2H, Sac–H), 4.49 (s, 1H, Sac–H), 4.38 (s, 1H, Sac–H), 4.01 (t, *J* = 4.2 Hz, 1H, Sac–H), 3.88 (t, *J* = 4.5 Hz, 1H, Sac–H), 3.78–3.70 (m, 1H, Sac–H), 3.46–3.06 (m, 3H, Sac–H), 2.52–2.43 (m, 2H), 1.97–1.88 (s, 1H), 1.52–1.48 (m, 2H), 1.22–1.18 (s, 26H), 0.99–0.93(m, 3H) 0.80 (d, *J* = 7.1 Hz, 3H). ^13^C NMR (75 MHz, CDCl_3_, Figure S4) *δ* = 169.5, 156.0, 129.8, 129.6, 122.7, 114.8, 111.6, 75.7, 74.3, 71.8, 68.6, 68.5, 67.2, 62.3, 35.9, 31.9, 31.7, 31.4, 29.7, 29.4, 28.9, 27.2, 22.7, 20.7, 20.6, 14.1. Broad signals were observed because of molecular self-assembly.

^1^H NMR spectrum of imine intermediate in CDCl_3_ was shown in Figure S6. The mass spectra of compound **5b** was shown in Figure S7.

### 4.4. Gelation Method

A known quantity of glycolipid was mixed with appropriate amount of solvent/oil in a sealed glass vial, and the entire content was heated until the solid was dissolved. The resulting solution was slowly allowed to cool slowly to room temperature, and gelation was visually observed by inverting the vial upside down. A gel sample that exhibited no gravitational flow in inverted vial was obtained and denoted as “G”. Instead of forming gel, if it remains as solution, it is referred to as “S” (solution) and if it remains as precipitate, then the system was denoted as “P” (precipitation). The system, in which the gelator is not soluble even at the boiling point of the solvent, was called an insoluble system (I).

### 4.5. UV-Visible and Circular Dichroism (CD) Spectral Analysis

UV-vis spectra were recorded on Thermo Scientific Evolution 220 UV/visible spectrophotometer (Waltham, MA, USA). The spectra were recorded in the continuous mode between 200 and 700 nm, with a wavelength increment of 1 nm and a bandwidth of 1 nm. CD spectra were obtained using JASCO J-815 CD spectrometer (Easton, MD, USA). The samples were loaded in a quartz cuvette of 0.1 cm path length. 

### 4.6. X-ray Diffraction Studies 

Small Angle X-ray diffraction (SAXRD) measurement for the xerogel formed from the compound **5b** was performed on a BRUKER-binary V3 diffractometer system.

### 4.7. Morphological Analysis 

Morphological feature of the hydrogel prepared from compound **5b** was studied using a JEOL JSM-6701F ultrahigh resolution field emission scanning electron microscope (FESEM) (JEOL, Tokyo, Japan) and high-resolution transmission electron microscopy (HRTEM) using JEOL JEM 2100 F HRTEM (JEOL, Tokyo, Japan).

### 4.8. Rheological Measurements

The mechanical behavior of the hydrogel formed by the glycolipid was investigated using a stress controlled rheometer (Anton Paar 302 rheometer, Anton Paar, Graz, Austria) equipped with a steel-coated 25 mm parallel-plate geometry. The gap between two plates for rheological testing of glycolipids was 1 mm and the experiments were carried out at 23 °C. Firstly, amplitude sweep measurement was conducted, which provides the information about linear viscoelastic range which is directly proportional to the mechanical strength of the gel material. Secondly, the measurement of the storage modulus, G′ and the loss modulus, G′′ were monitored as functions of frequency from 0.1 to 300 rad s^−1^.

## Figures and Tables

**Table 1:** Gelation ability of glycolipids, **5a** and **5b** in various solvents/oil.

S.No.	Solvent/Oil	Observation ^a^ (CGC % *w*/*v*)	
		5a	5b
1	Cyclohexane	S	S
2	Toluene	S	S
3	1,2-Dichlorobenzene	S	S
4	DMSO	S	S
5	DMF	S	S
6	DMSO + Water (1:5)	PG	G (0.67)
7	DMF + Water (1:5)	PG	G (1.5)
8	Heavy paraffin Oil	PG	PG
9	Heavy paraffin Oil	PG	PG
10	Hazelnut Oil	S	S
11	Linseed oil	PG	PG
12	Methanol	S	S
13	Methanol + water (1:5)	S	G (0.67)
14	Ethanol	S	S
15	Ethanol + water (1:5)	PG	G (0.29)
16	Water	I	I
17	Decanol	PG	PG
18	Dodecanol	PG	PG

^a^ G: Gel; PG: Partial gel; S: Solution; P: Precipitate; I: Insoluble. Critical gelator concentration (CGC) is presented in parenthesis (% (*w*/*v*)). DMSO: Dimethyl sulfoxide; DMF: *N,N*-Dimethylformamide.

## Supplementary Materials

The following are available online at www.mdpi.com/2310-2861/4/1/1/s1.
