# Supplementary material for: Supramolecular Gel Formation Based on Glycolipids Derived from Renewable Resources
Source: Gels. 2017 Dec 24;4(1):1. doi: 10.3390/gels4010001 (PMC6318777; doi:10.3390/gels4010001)
Supplement: Supplementary file 1 [file gels-04-00001-s001.pdf]

Supramolecular Gel Formation Based on Glycolipids Derived from Renewable Resources

Krishnamoorthy Lalitha, Kandasamy Gayathri, Yadavali Siva Prasad, Rajendhiran Saritha, A. Thamizhanban, C. Uma Maheswari, Vellaisamy Sridharan and Subbiah Nagarajan

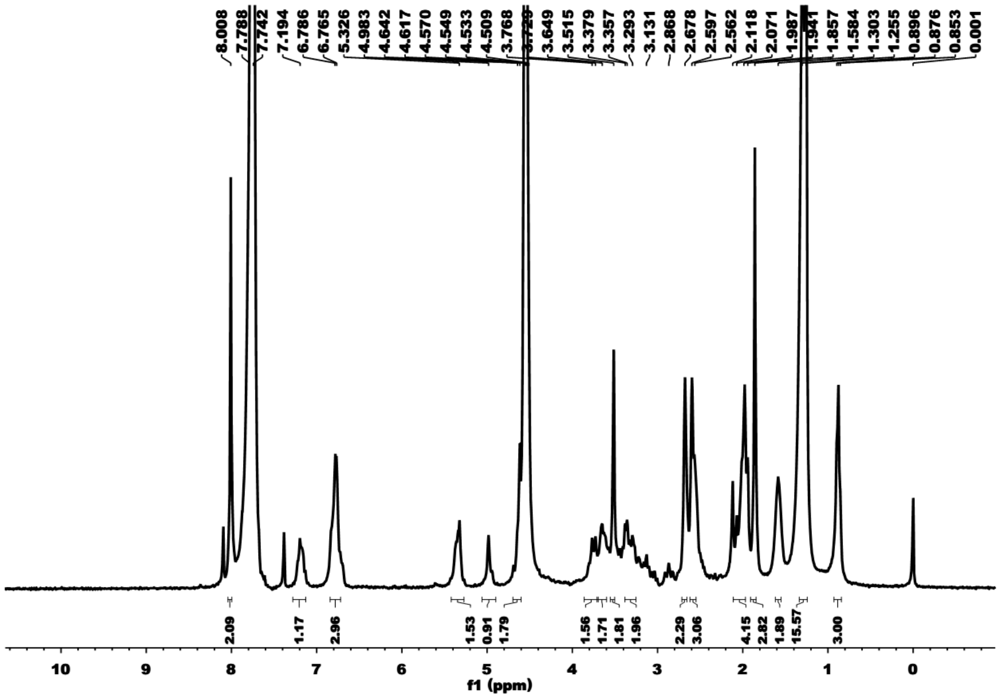

Figure S1. ^{1}H NMR spectrum of compound **5a** in DMSO-d_6

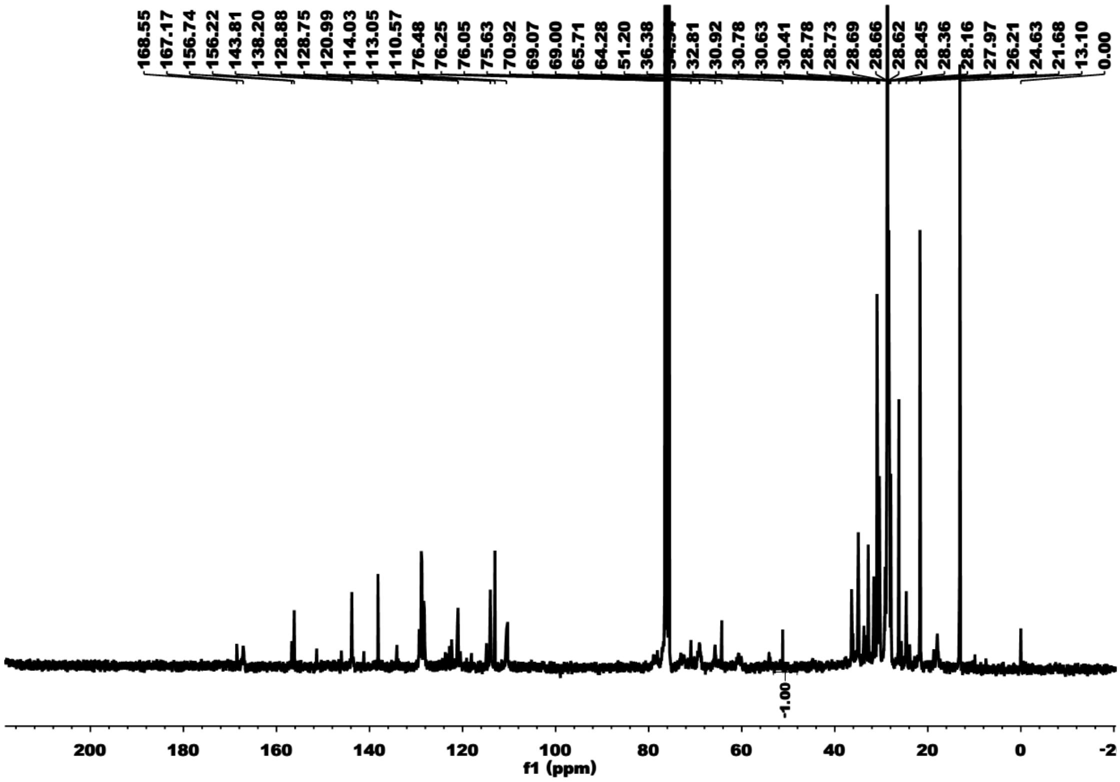

Figure S2. ^{13}C NMR spectrum of compound **5a** in DMSO-d_6

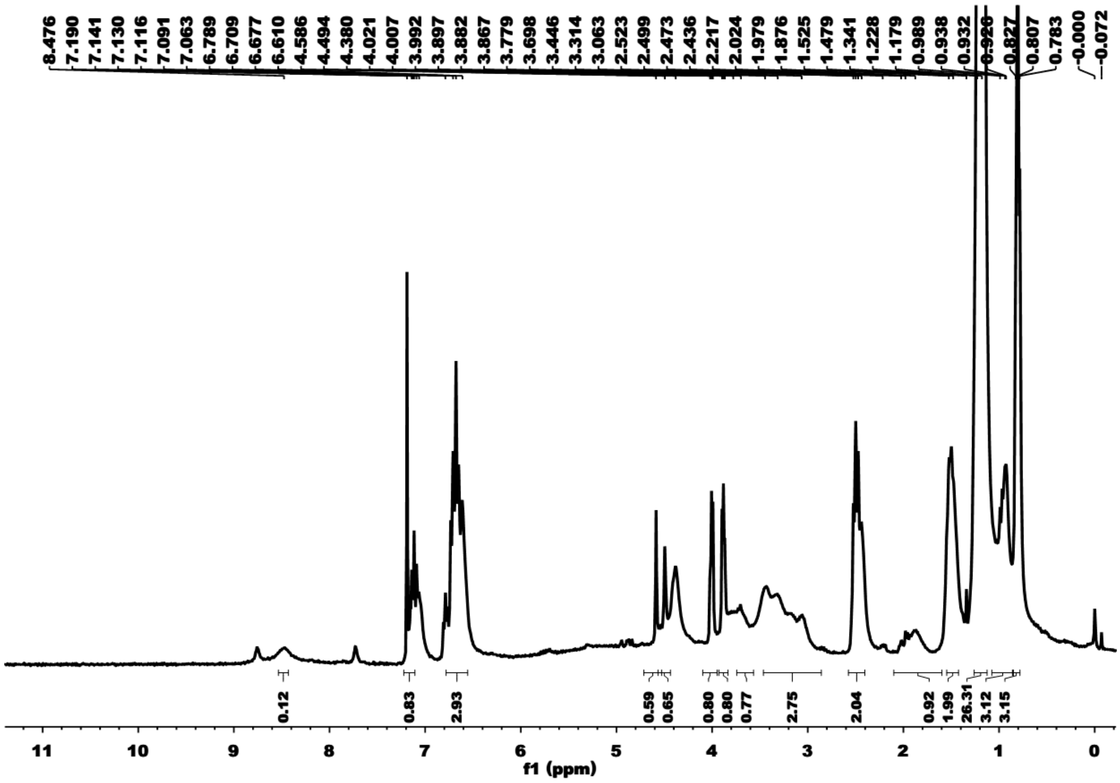

Figure S3. ^{1}H NMR spectrum of compound **5b** in $CDCl_3$

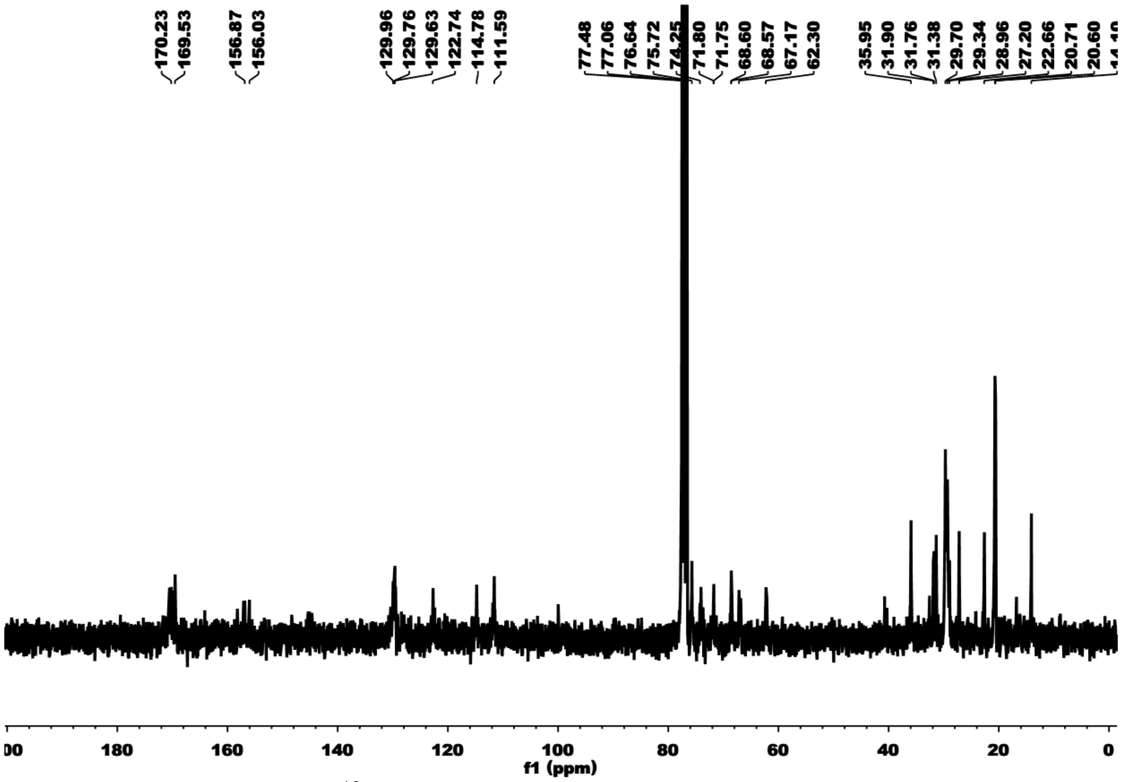

Figure S4. ^{13}C NMR spectrum of compound **5b** in $CDCl_3$

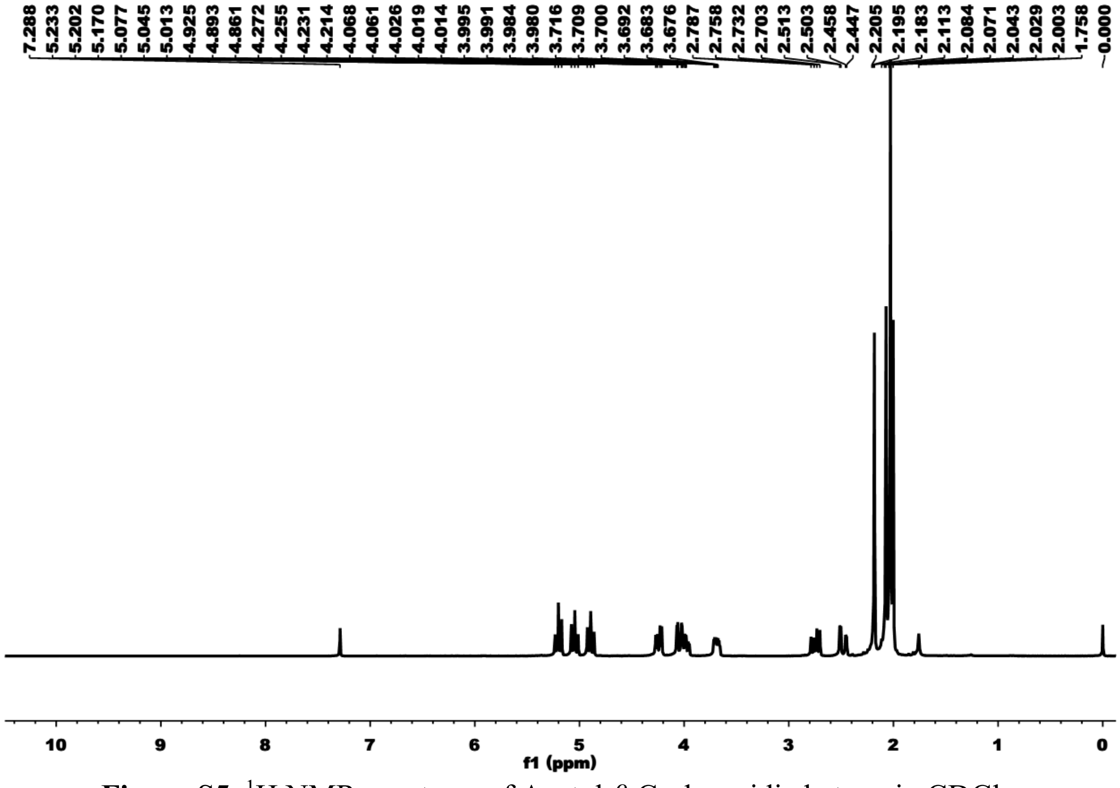

Figure S5. ^{1}H NMR spectrum of Acetyl β-C-glycosidic ketone in $CDCl_3$.

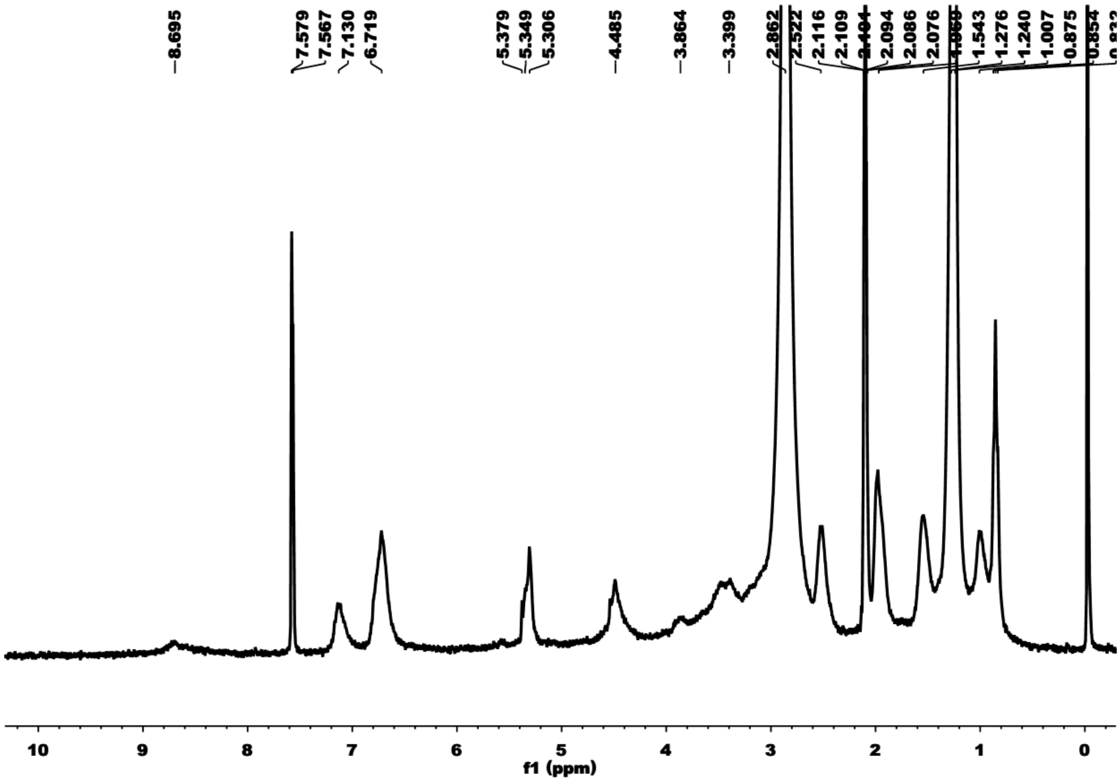

Figure S6. ^{1}H NMR spectrum of imine intermediate in $CDCl_3$.

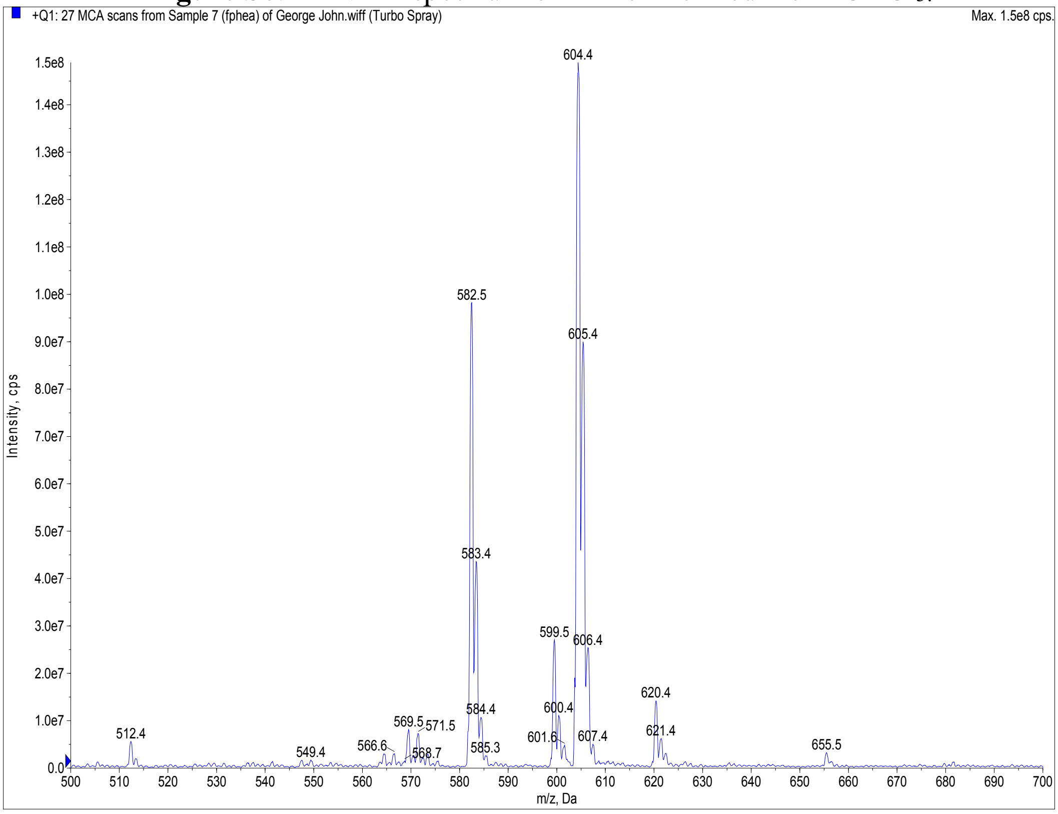

Figure S7. Mass spectra of compound 5b.
